# Mantle plume trail beneath the ca. 1.1 Ga North American Midcontinent Rift revealed by magnetotelluric data

**DOI:** 10.1093/nsr/nwae239

**Published:** 2024-07-13

**Authors:** Wule Lin, Adam Schultz, Bo Yang, Lyal B Harris, Xiangyun Hu

**Affiliations:** Hubei Subsurface Multi-scale Imaging Key Laboratory, School of Geophysics and Geomatics, China University of Geosciences, Wuhan 430074, China; Department of Ocean Science and Engineering, Southern University of Science and Technology, Shenzhen 518005, China; Advanced Institute for Ocean Research, Southern University of Science and Technology, Shenzhen 518005, China; College of Earth, Ocean, and Atmospheric Sciences, Oregon State University, Corvallis, OR 97331, USA; Key Laboratory of Ocean and Marginal Sea Geology, South China Sea Institute of Oceanology, Chinese Academy of Sciences, Guangzhou 511458, China; China-Pakistan Joint Research Center on Earth Sciences, CAS-HEC, Islamabad 45320, Pakistan; Eau Terre Environnement Research Centre, Institut National de la Recherche Scientifique, Québec, QC G1K 9A9, Canada; Hubei Subsurface Multi-scale Imaging Key Laboratory, School of Geophysics and Geomatics, China University of Geosciences, Wuhan 430074, China

**Keywords:** magnetotelluric, Midcontinent Rift, Keweenaw mantle plume, Superior Province, North America

## Abstract

Whilst the 1.1 Ga North American Midcontinent Rift (MCR) system is formed in association with the Keweenaw mantle plume, the absence of a northern third rift arm or aulacogen (a general characteristic of mantle plumes) has previously not been well understood. To help clarify this unusual plume–rift relationship and to better establish the region affected by the Keweenaw mantle plume, we present the first electrical resistivity model of the MCR derived from 3D inversion of EarthScope USArray and Lithoprobe magnetotelluric (MT) data, extending northwards into the Archean Superior Province. Our model shows a prominent highly conductive anomaly trending NW-SE at the base of Western Superior's cratonic lithospheric mantle, cross-cutting and extending for over 300 km on both sides of the western rift branch. We propose that this anomaly reflects the ancient signature of a plume trail, resulting from metasomatism and/or partial melting of the sulfide-rich basal lithospheric mantle during impingement of the Keweenaw mantle plume.

## Teaser

Geophysical evidence in the Lake Superior region reveals the deep trail left by ancient North America's past rapid plate motion over a mantle plume.

## INTRODUCTION

**Figure 1.**
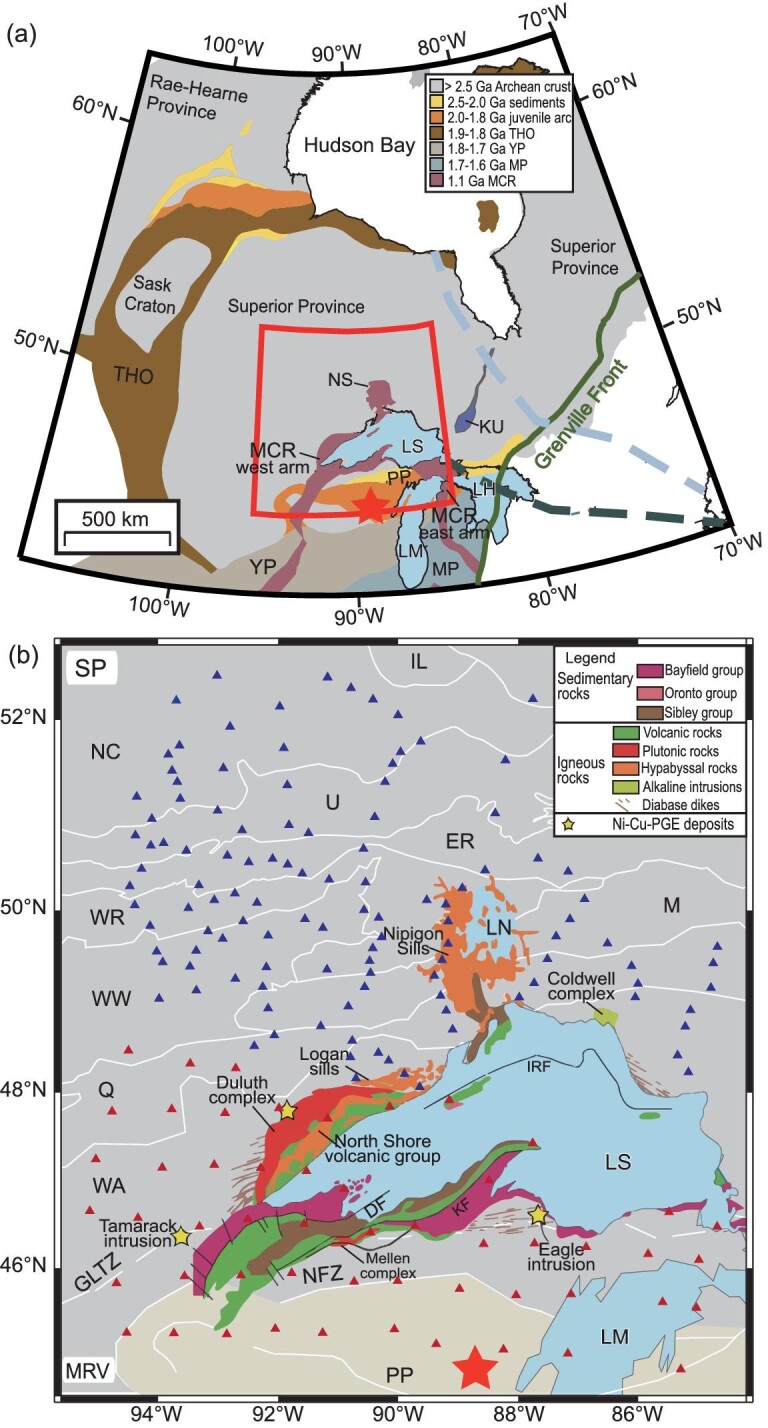
(a) Simplified tectonic map of central North America [5] with the red box indicating our study area, as shown in (b) with more detail. The light steel blue and dark slate gray thick dashed lines represent the Great Meteor hotspot track inferred by kimberlite occurrences [6] and seismic evidence at a depth of 200 km [7], respectively. The red star represents the interpreted Keweenaw mantle plume center [8]. (b) Regional geological map of the study area with white lines showing the terrane boundaries [9,10] as well as MCR-related intrusions. The red and blue triangles represent the EarthScope and Lithoprobe MT stations used in this study, respectively. Abbreviations: MCR = Midcontinent Rift (purple shaded area); NS = Nipigon Sill; THO = Trans-Hudson Orogen; PP = Penokean Province; YP = Yavapai Province; MP = Mazatzal Province; KU = Kapuskasing Uplift; IL = Island Lake; NC = North Caribou; U = Uchi; ER = English River; WR = Winnipeg River; WW = West Wabigoon; M = Marion; Q = Quetico; WA = Wawa-Abitibi; MRV = Minnesota River Valley. IRF = Isle Royale Fault; DF = Douglas Fault; KF = Keweenaw Fault; GLTZ = Great Lakes Tectonic Zone; NFZ = Niagara Fault Zone. LS = Lake Superior; LM = Lake Michigan; LH = Lake Huron.

The Midcontinent Rift (MCR), or Keweenawan Rift, one of the prominent Precambrian geologic features of central North America, is a failed 1110–1085 Ma rift formed within the Archean Superior Province of Laurentia (Fig. 1). The MCR, which contains enormous volumes of igneous rocks outcropping and in the subsurface near Lake Superior, has been explained by a combination of reactivation of pre-existing structures and the upwelling and decompression melting associated with the Keweenaw mantle plume [1,2], or possibly by anomalously hot or fertile upper mantle upwelling [3]. Its surface geometry, interpreted from potential field data and regional geology [4], portrays two intersecting rift arms that cut across Archean and Paleoproterozoic terrains; the west arm extends southwestwards for ca. 2000 km and the east arm is truncated by the Grenville orogen [5–10].

The MCR contrasts with the classical, intersecting three-arm model of continental rifting above a mantle plume, where even if not fully developed, an aulacogen or failed rift constitutes a third arm [11]. Whether and to what extent the Superior craton was affected by the MCR plume northwards of the interpreted plume center has met with some controversy. The 1117–1106 Ma mafic to ultramafic intrusions and 1114–1110 Ma Nipigon diabase sills (where the Nipigon embayment is underlain by low velocity lithospheric mantle [12]) overlap with the early stages of the MCR. However, older faults control emplacement of Keweenawan Supergroup intrusions and extensional features [13] and the absence of a velocity anomaly associated with underplating [14] provides little evidence for a third arm/aulacogen, and no deep third arm is shown by Bouguer gravity [3].

**Figure 2.**
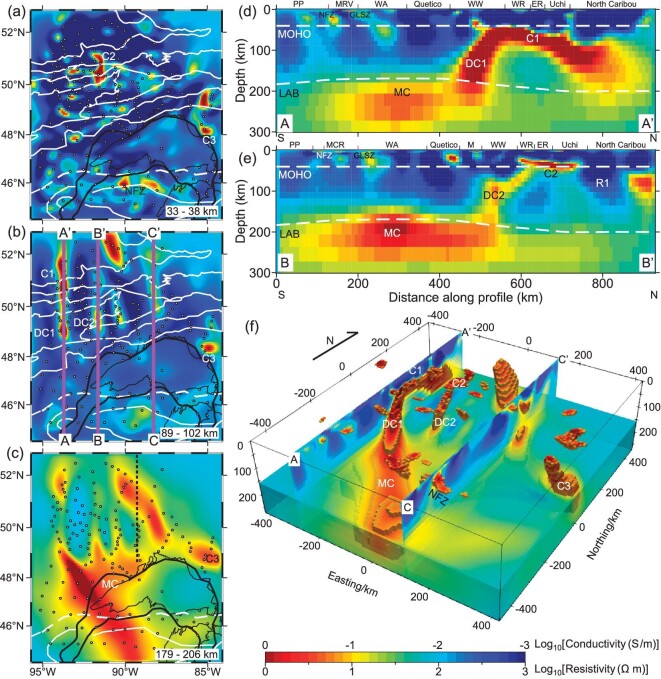
Preferred resistivity model derived from MT 3D inversion. (a–c) Horizontal slices at depths of 33–38 km, 89–102 km and 179–206 km. Black circles denote MT stations, the MCR is outlined by a thick black solid line and subprovince boundaries are shown as white lines (c.f. Fig. 1). The black dashed line in (c) illustrates the western boundary of the East Superior low-velocity zone [29,30]. (d and e) Vertical cross-sections A–A’ and B–B’, whose locations are shown in (b). The dashed white lines show the estimated seismic Moho and LAB [25,26], which are in good agreement with our established electrical structure. PP = Penokean Province; MRV = Minnesota River Valley; WA = Wawa-Abitibi; WW = Western Wabigoon; WR = Winnipeg River; NFZ = Niagara Fault Zone; GLTZ = Great Lakes Tectonic Zone; M = Marmion. (f) A perspective view of low resistivity anomalies (≤10 Ω m) below 20 km depth. The location of cross-sections A–A’ and C–C’ are shown in (b). C denotes a conductive body and R denotes a resistive body.

The electrical resistivity (or its reciprocal, conductivity) structure derived from magnetotelluric (MT) investigations has been widely used to study the structure and evolution of the tectonically stable cratons [15], including the MCR area where data from the EarthScope MT array [16] have provided quasi-uniform coverage of the well-established arms of the MCR [17–19]. In particular, a prominent northwest elongated conductive feature at the base of lithosphere near western Lake Superior was previously identified and related to the Keweenaw mantle plume [19]. In order to seek a potential northern rift arm and better constrain and map the continuation of this conductive feature outside of the area covered by EarthScope MT data, Lithoprobe MT data in Canada were also used in the present study (red and blue triangles in Fig. 1b). Depth slices and a 3D electrical resistivity model beneath the North American Midcontinent were constructed (Figs 1 and 2) using both the EarthScope and Lithoprobe MT data to better understand the structure and origin of the MCR and related features. As we will show, our model illustrates that the prominent high conductivity anomaly previously identified further south [19] continues beneath the southwestern Superior Province from the upper mantle to the lower crust, which is interpreted to relate to its southwards passage over the Keweenaw mantle plume.

## GEOLOGICAL SETTING

The MCR and most of our study area to the north lies within the Superior Province (Fig. 1), on whose southeastern boundary accretion of the Penokean Province along the Niagara Fault zone took place at 1.9–1.8 Ga [10]; accretion of the Yavapai Province at 1.8–1.7 Ga and the Mazatzal Province at 1.7–1.6 Ga [5] followed. The final, collisional stage of the Grenville orogeny between 1.08 and 0.98 Ga in the adjoining Grenville Province is thought to have stopped the active phase of rifting of the MCR due to the compressional stress produced (although other explanations have also been proposed [4]). Nevertheless, no significant post-Grenvillian tectonic events affected the MCR, making it possible to explore its older signature(s).

The traditional model for the western Superior Province [20] posits the north to south assembly of generally east-west trending ribbon terranes (including Eoarchean to Mesoarchean fragments) and subduction-related arcs and back arc basins, progressively accreted to the Hudson Bay Terrane (the southern portion of the Eoarchean to Mesoarchean northern Superior proto-craton). In this actualistic plate tectonic model, the final event was the accretion of the southernmost, Minnesota Valley River terrane along the Great Lakes Suture Zone [9,20] (Fig. 1). Other, non-plate tectonic models for Archean tectonics [21] applied to the Superior Province [22–24] involve rifting and fragmentation of the Hudson Bay Terrane and subsequent closure and regional shortening due to the southwards displacement of the northern Superior proto-craton, driven by mantle flow against its deep lithospheric keel. Whether or not fossil subduction zones and sutures were present in the two tectonic models respectively has implications for interpreting the source(s) of MT anomalies in our study areas, which are discussed later.

## RESULTS

Details of the MT data and inverse modeling are given in the Methods section below, while related phase tensor analysis is shown in the supplementary data (Fig. S1). Figure 2 presents map views of the 3D resistivity model at selected representative depths, vertical cross-sections across notable features, and a 3D view of the high conductivity zone within our preferred inversion model. These results show:

- Scattered low and high resistivity anomalies at lower crustal (33 km to 38 km) depths, including conductive anomalies C2 and C3, which are roughly aligned N-S and E-W, except for the SE area of the map where they trend NW-SE (Fig. 2a).
- N-S trending anomalies at upper- to mid-lithospheric mantle depths of 89–102 km in the central to NW regions, N of the Quetico Subprovince, and the isolated C3 conductive anomaly close to the NE shore of Lake Superior.
- A NW-SE trending high conductivity anomaly (MC) at depths of 179–206 km is shown in Fig. 2c. A detailed study shows lithospheric mantle thickness (i.e. ‘LAB depth’) in this part of the Superior Province ranges between 140 and 180 km (increasing to between 200 and 220 km in the northernmost part) [25]. Other more regional seismic LAB estimates are 150–190 ± 7 km [26] and 185–220 km [27], and the petrological-based LAB depth has been calculated as being between 150 and 175 km [28]. Slices through a 3D model [23] show that our study area is situated in a wide sub-continental lithospheric mantle (SCLM) rift between two older terrains with deep lithospheric keels (viz. the Hudson Bay and Minnesota River Valley subprovinces). Therefore, these anomalies shown on this depth slice have, for all or for the most part, a source beneath the SCLM. The linear, higher conductivity NW-SE trending portion of anomaly MC is a continuation of the aforementioned feature shown in [19], and passes beneath the west arm of the MCR in the southwestern narrow part of Lake Superior, ca. 400 km from its intersection with the east MCR arm. A broader and more diffuse extension of this anomaly follows the NW shore of Lake Superior from which two short, eastern apophyses extend in a more northerly direction.
- In 3D, two stem-shaped, sub-vertical conductors (DC1 and DC2) rise through the SCLM from ∼150 km depth to the Moho, beneath which conductor DC1 forms a broad arch (Fig. 2d) or directly upon which a sub-horizontal conductive anomaly occurs (DC2, Fig. 2e). Two shallow high conductivity bodies (C1 and C2) rise from anomalies DC1 and DC2 in the deep crust. Importantly, these features are not largely affected by various inversion parameters (Figs S4–S8) and were well tested (Figs S9–S11), suggesting the robustness of these high conductivity bodies. The deepest mantle anomalies in Fig. 2c terminate at approximately the same position within the Western Wabigoon subprovince from which the 89–102 km deep anomalies in Fig. 2b rise.

The prominent high N-S conductivity anomalies within the lower crust and upper mantle are unusual in the stable cratonic area characterized by high-velocity anomalies [29–31] as well as relatively thick lithosphere [25,26] (although shallower than in the northern Superior proto-craton/Hudson Bay Terrane further north). The geoelectric strike direction obtained from MT data (Fig. S1) above the conductive features (Fig. 2b) revealed an obvious N-S trend, especially at periods centered near 528 s that are sensitive to the depth of the lithospheric mantle. The somewhat greater variability of strike directions at short periods might be related to the complexity of shallow structures (e.g. Fig. 2a), and the northwestern trend at longer periods (e.g. centered near 4673 s in Fig. S1) correlates with the structure at great depth. The MT responses calculated from the preferred model (full-impedance tensors and tipper vectors) fit the observed data well, with a normalized root-mean-square misfit of 2.33 for a 5% error floor for impedance and a constant 0.05 for tippers. We thus conclude that N-S alignment of the conductive features is a robust feature of the data set.

The 400 km long and 50 km wide arch-shaped highly conductive N-S feature (C1-DC1) in the SCLM along profile A–A’ has an extremely low resistivity of ∼1 Ω m. Given the possible screening effect of the shallow high conductivity features, we carried out a model sensitivity test of DC1 (Fig. S10) that confirmed its validity. Other conductive features of similar geometry but smaller size (C2 in the lower crust and possibly uppermost mantle and DC2 in SCLM) are shown in section B–B’. The conductivity of DC1 is higher than that of DC2.

## DISCUSSION

### Timing and significance of MT anomalies

Although cutting the western arm of the MCR, ca. 400 km SW of the intersection of the rift's two arms, the long NW-SE trending, highest conductivity section of anomaly MC directly underlies the interpreted Keweenaw mantle plume center [8] (red star in Fig. 1b). This anomaly also underlies: (i) mafic-ultramafic and anorthosite intrusions of the 5000 km^2^, 1096 Ma Duluth intrusive complex (one of the largest on Earth), (ii) similar mafic intrusions such as the Mellen Complex on the opposite, SE side of Lake Superior, and (iii) coeval flows of the North Shore Volcanic Group (Fig. 1b), which are all attributed to multiple magmatic pulses associated with lithospheric extension and decompression melting above a zone of mantle upwelling [32,33]. Along its SE continuation [19], the 12-km-long Round Lake mafic–ultramafic intrusion, mafic dikes of a 1.0 Ga swarm, and anorogenic granitoids and volcanics closer to Lake Michigan occur. The ferrogabbro Logan sills and the 1105.6 ± 1.2 Ma, Ni–Cu–PGE sulfide-bearing Tamarack intrusive complex [34] are both situated in a broader extension of this anomaly along the shore of Lake Superior and 75 km west of Duluth, respectively (Fig. 1b). The 1.1 Ga Heaven Lake mafic-ultramafic intrusion, which hosts Ni–Cu–Co–PGE mineralization, occurs in the easternmost of the two additional diffuse conductive anomalies arising from the broad portion of the MC anomaly. Previous geophysical [35] and thermochronological [36] studies support that the high conductivity anomaly MC lies within the footprint of the area which was influenced by the Keweenaw mantle plume.

**Figure 3.**
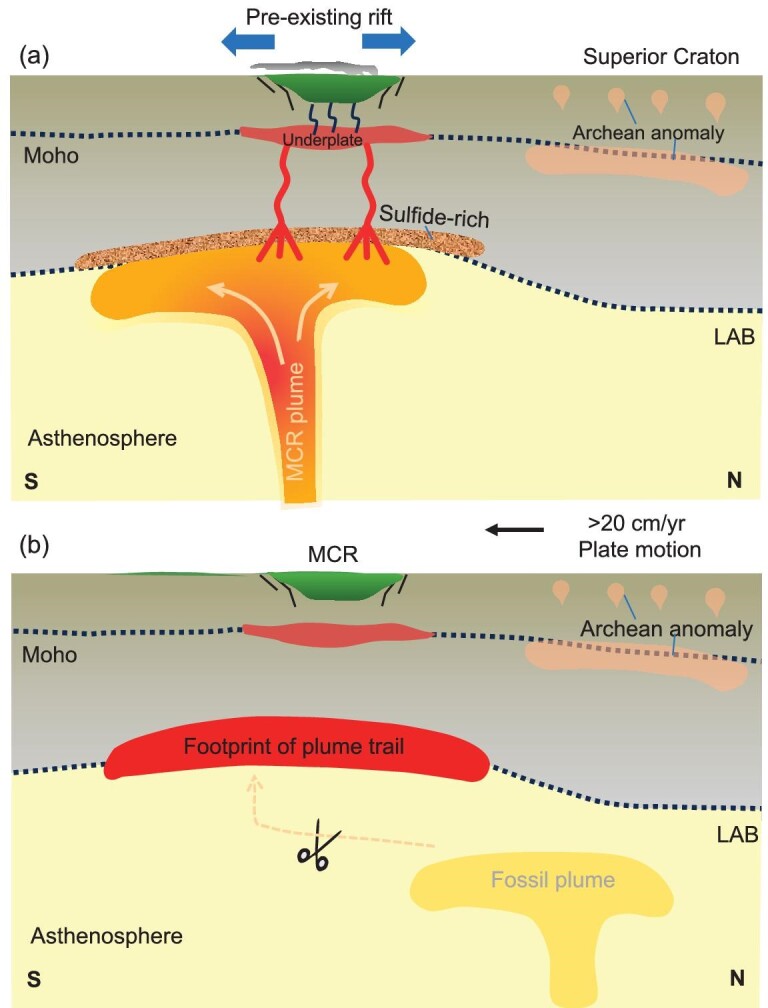
Possible model for the formation of the conductive anomalies at the base of the lithosphere around Lake Superior. Volcanics are shown in green. (a) A segment of the Superior Province has sulfide-rich lithosphere [19]. The Keweenaw mantle plume's impingement on the SCLM around Lake Superior provides a potential mechanism to form interconnections between sulfidic films, yielding the observed highly conductive features at great depths. Pre-existing rift regions [3] are reactivated during plume impingement, localizing flood basalt magmatism, underplating, mafic-ultramafic intrusions and sulfide-rich mineral deposits around Lake Superior. Several MT anomalies within the Superior are likely due to Archean hydrothermal fluid flow and magma emplacement. (b) The >20 cm/yr southward (in today's orientation) rapid motion of Laurentia during the period of MCR formation [38] severed the connection to the plume, leaving the regional NW-SE trending conductive trail.

When rotated to match the orientation of the MCR within Laurentia at 1108 Ma [37], this linear, NW-SE MC conductive anomaly at the base of the lithosphere parallels the direction of Laurentia's rapid southward displacement (exceeding 20 cm/yr) determined from paleomagnetic data [38]. As interpreted from thermobarometric models, the magma generation mechanism for MCR basalt changed from early mantle plume-related melting to subsequent decompressional melting controlled by mantle convection [39], implying that this rapid plate motion may have broken the connection to the mantle plume beneath the pre-existing structures which created the initial MCR (Fig. 3b), only leaving a resistive trail with little evidence for magmatism and volcanism NW of the MCR. That the MT anomaly does not extend further northwestwards than the Western Wabigoon subprovince may be due to a difference in the composition of SCLM (such as a higher sulfide content), commensurate with a change in age from late Paleoarchean to Mesoarchean to the north, to late Mesoarchean to Neoarchean to the south [40] of this lithospheric-scale boundary. The concentration of magmatic sulfide deposits and occurrences in the area traversed by this anomaly and its SE termination at the Superior-Yavapai contact likely reflects a unique, sulfide-rich composition of this segment of the Superior Province.

Interpretation of other conductive MT anomalies is less straightforward, and there are three possible options:

***Option 1***. In this first option, all MT anomalies in our study area are due to a regional, up-to-1000-km-diameter thermal footprint about the Keweenaw mantle plume center [36,41] (including that of a suggested older, 1150 to 1140 Ma precursor event [41]). The subvertical conductors DC1 and DC2 (Fig. 2) are here thought to represent the fossil signatures of the ascending channels that transported the fluids from the asthenosphere into the SCLM and lower to mid-crust, forming anomalies C1 and C2. This requires the presence of pre-existing structures at a high angle to those mapped at the surface, where only few ca. N-S structures are present.

In the conventional plate-tectonic model for the Superior Province, seismic reflection, refraction and gravity models suggest the presence of a preserved slab of accreted oceanic crust and upper mantle subducted northwards from beneath the central Wabigoon subprovince (Fig. 1b) [42,43]. Such sutures elsewhere are most often imaged as conductive anomalies on MT surveys due to graphite having been carried deep into the Earth during subduction and transported by fluids into associated crustal shear zones during orogenesis [18,44,45]. Although in cross-section, anomaly C1 is coincident with the interpreted fossil subduction zone in this model, in plan and 3D view anomalies C1 and C2 cut across the interpreted sutures corresponding to subprovince boundaries (Figs 1b and 2). Whilst direct imaging of an Archean subducted slab is not tenable, fluids or melts that ascended along DC1 may have been impeded by the subducted depleted mantle interface and moved laterally along the remnant slab, rising where breached by deep transverse N-S faults. During this time, at least some hydrothermal fluids or melt, or mixtures thereof, with sulfide-enriched content were left behind, or host rocks were metasomatized to form the prominent high conductivity zones.

In this model, the NW-SE trending conductive anomaly beneath or in the lowermost of the SCLM intersecting the two arms of the MCR near the northern extremity of Lake Superior (Fig. 2) may represent an incipient, third arm of a classic plume-related failed rift/aulacogen [11]. This would, however, require that either the uppermost plume head comprised two lobes or that magma migrated laterally into pre-existing structures [46,47].

***Option 2.*** In this second option, only the NW-SE conductive anomaly at the base of the lithosphere and its broader extent on the NW margin of Lake Superior, anomaly MC, formed during displacement of the Superior Province over the Keweenaw mantle plume (given the arguments presented in detail above for a mantle plume-related origin), but the anomalies higher in the SCLM and crustal anomalies instead formed in the Archean and were not modified (or at least not substantially) by the thermal effects of the Keweenaw mantle plume. Although still plausible in the context of subduction-accretion tectonics conventionally ascribed to the Superior Province, this model can be best explained by the fragmentation and reassembly interpretation for the Superior Province without modern plate tectonics [22–24] (i.e. a model without fossil subduction zones that may contribute to the formation of anomalies described above for *Option 1*, but without the orientation constraints). In this model, as suggested based on a preliminary analysis of only part of Lithoprobe MT data in the northern part of the present study area [48], pre-existing structures at a high angle to the generally E-W subprovince boundaries in the Western Superior, i.e. akin to those in the NE Superior Province, preserved in Paleo- to Mesoarchean SCLM (especially north of the Western Wabigoon and Quetico subprovinces [40]), may have controlled circulation of hydrothermal fluids and possibly magma, giving rise to the N-S conductive anomalies DC1 and DC2 in the SCLM (Fig. 2b). From these broad N-S conductive anomalies in SCLM, fingers (C2 and smaller anomalies west of it aligned N-S) have risen through the crust (Fig. 2a) similar to the ‘Fingers of God’ in the Gawler Craton of South Australia [49]. Indeed, similar zones of attenuation on reflection seismic (a.k.a. ‘bland zones’) that are comparable to MT ‘Fingers’ mentioned in the article on the Gawler Craton [49] have been identified in the NW Superior Province [48].

Other isolated anomalies such as C3 that rise from the SCLM into the crust may be due to more local controls of Archean structures; although located close to the MCR, anomaly C3 is still a considerable distance from the interpreted plume center and NW-SE interpreted trail left by passage over the mantle plume. The central to eastern Superior Craton is interpreted to have transited over a Great Meteor hotspot during the Jurassic and Early Cretaceous [6] (although such a hotspot model is contested [50]). Figure 1a shows the track of this hotspot at the surface inferred from kimberlite pipes [6] and at a depth of 200 km from seismic evidence [7]. Both tracks are outside of our study area, suggesting this hotspot has no significant influence on the main conductors we focus on, which thus preserved the tectonic footprint of the MCR. However, the conductor C3 at the western end of the track (dark slate gray) might be associated with this hotspot, which is consistent with an observed low-velocity anomaly [5,7,29].

***Option 3.*** A third alternative is that all the MT anomalies in the study area formed in the Archaean and that either no anomalies were created by the Keweenaw mantle plume or that there was no plume and that magmatism in the MCR was controlled solely by asthenospheric uplift during rifting. Whilst still possible, solid arguments were presented under *Option 1*, above, for the formation of the conductive anomaly in the basal SCLM or underlying it during southward displacement of the Superior Province (as part of Laurentia) over a mantle plume. That there was no mantle plume is far less likely (and is noted here solely for completeness), given the aforementioned conjuncture between the intrusion of large mafic bodies, extensive volcanism and both radial and circumferential dikes characteristic of a mantle plume [8].

### Origins of conductivity anomalies

Highly conductive zones within the lithosphere are generally explained by the presence of partial melt or saline fluid, by interconnected graphitic or sulfide mineral-bearing zones, or by the presence of hydrogen in mantle olivine, which along with pyroxene and garnet accommodates water in the upper mantle. Considering the stable Precambrian geological setting along with the low heat flow of our study area [51], extant partial melt and saline fluid are unlikely to explain these prominent high conductivity anomalies [15]. Therefore, hydrous minerals and/or sulfide-bearing minerals are the more likely cause of the highly conductive asthenospheric and lithospheric features in this study. Additionally, conductivity anomalies in the stable lithosphere above 150 km deep may possibly be explained by grain boundary graphite films [52,53].

For the extremely high conductors C1, C2 and DC1 within the SCLM, the inferred water content from any law of hydrous olivine electrical measurements in the laboratory [54] would induce melting in the lithospheric mantle [55]. The implausibly high water content of ∼800 ppm is required to explain DC2 (ca. 10 Ω m) using the unified electrical conductivity law [54], a level that could also induce melting [55], yet we cannot rule out more typical levels of mantle hydration (50–200 ppm) [55]. Nevertheless, even for such levels of hydration, other connected conductive phases must also be present.

Interconnected graphite films along mineral grain boundaries have been widely used to explain the high conductivity in the stable cratonic lithosphere, particularly along sutures and terrane boundaries [15,44,45]. If the conductive anomalies are dominated by the interconnected graphite with a typical resistivity of 0.1 Ω m, an unlikely large amount of graphite >10 vol% is required to explain the extremely high conductivity (i.e. 1 Ω m), while for a resistivity of 10 Ω m, the graphite required would be reduced to 1 or 1.5 vol% (Fig. S12). A similar content of graphite was used to explain the observed highly conductive anomaly in an adjacent region [45].

It is important to note that the resistivity of graphite could be very low (e.g. 5 × 10^−6^ Ω m, i.e. 2 × 10^5^ S/m) yet capable of producing the bulk resistivity of 10^−3^ Ω m with a volume of 1 vol% provided the graphite films have bulk interconnections along grain boundaries [52]. This might be the reason that various studies attribute high conductivity lithospheric anomalies (e.g. 1 Ω m) to graphite without considering its content. For the depths and pressures associated with the deep lithospheric anomalies in our preferred model, graphite is unstable and transforms into highly electrically resistive diamond structure at sub-cratonic depths of ∼150 km [53], suggesting that the presence of interconnected sulfides is a more likely cause of enhanced conductivity, particularly for the deep part of DC1.

Massive sulfide deposits in the Lake Superior region [56,57] occur within mafic-ultramafic intrusions with an SCLM source [58]. Sulfide was suggested to be able to enhance conductivity by laboratory measurements [59,60]. It was used to interpret the high conductivity anomaly in cratonic environments such as in the mantle beneath the Kaapvaal craton [61]. The conductivity of the solid sulfides is very high (10^3^–10^5^ S/m) [62,63], and at most 0.1 vol% sulfide would account for the bulk conductivity of 1 S/m (Fig. S12), providing it forms a well-interconnected network. Such a scenario is not typical, however, because of the small fraction of sulfides in the mantle, i.e. typically 0.06 wt% [64].

As shown in Fig. 2, our model reveals widespread conductors (MC) with upper boundaries of ∼150–180 km, i.e. immediately below or at the base of the SCLM. Previous studies using EarthScope MT data alone also showed similar conductive features [18,19], although with limited data coverage. The presence of elevated conductivities across a wide area at the base of the lithospheric mantle that are comparable to conductor MC in our study has been previously noted elsewhere [65]; establishing their origin, however, remains problematic [66]. In particular, this elongated MC conductive feature was further tested before, with resistivity of ∼1–3 Ω m [19]. Similar sensitivity tests illustrate that MC starts from the asthenosphere (Fig. S11) with unresolved vertical extension (Fig. S9) due to the limitation of the data set.

Partial melting of the basal lithospheric mantle over a mantle plume with metasomatism by fluids or melts enriched in incompatible elements [15] is applicable to the regional context proposed for passage of the MCR over the Keweenaw mantle plume. Such metasomatic processes and refertilization of the lower lithospheric mantle above a subducting slab are also postulated to explain a regional deep SCLM conductive anomaly in the SW Gawler Craton of South Australia [66]. This anomaly's orientation at a high angle to the interpreted subduction zone makes a subduction-related origin unlikely, whereas metasomatism due to mantle dripping, with commensurate multiple zones of asthenospheric upwelling, also proposed for the Gawler Craton [67] following subduction, would provide a more likely mechanism, and be akin to displacement over a mantle plume. To interpret this high conductivity, at least 2000 ppm water content [68] that would induce melt, or 2% melt [69] are required, as have been discussed before [70]. This high melt fraction is not supported by the seismic results [29,31]. In addition, a much smaller carbonate melt would account for this anomaly [71]. However, during the plume or other large-scale melting events, carbonate melt would not always be well connected when both silicate and carbonate melts are present because the silicate melt preferentially wets the solid mantle matrix [72]. Importantly, partial melt would not be sustainable because of heat loss over such a long geological time within the craton.

The Keweenaw mantle plume's impingement on the SCLM around Lake Superior provides a potential mechanism to form bulk interconnections between sulfidic films, yielding the observed highly conductive features at great depths (Fig. 3a). Furthermore, Ni and Cu sulfides are most likely, due to the presence of Ni-Cu orebodies in the study area [34]. Evidence for the mantle plume is supported by other geophysical, geochemical and petrological studies [2,11,73]. Seismic studies observed a low-velocity channel down to ca. 250 km that is similar in geometry and near (but southwest) of the highest high conductivity of MC (Fig. 2c and f) [30], which may be related to the preserved track of a mantle plume [30]. A recent geodynamic model also demonstrated that a mantle plume is required to produce a large volume of flood basalt in the MCR [46].

## CONCLUSION

The first electrical resistivity model of the MCR derived from 3D inversion of both USArray and Lithoprobe MT data, extending northwards into the Archean Superior Province, shows a prominent highly conductive anomaly trending NW-SE at the base of the western Superior's cratonic lithospheric mantle, cross-cutting and extending for over 300 km on both sides of the west rift branch, and continuing on strike direction into an area of previously processed EarthScope MT data. The Keweenaw plume center interpreted in earlier studies, the penecontemporaneous Duluth and other layered mafic intrusive complexes hosting massive sulfide deposits, flows of the North Shore Volcanics, and a cluster of mafic dikes overlie different parts of this continuous conductive anomaly. We hence propose that the total, ca. 600-km-long anomaly, starting at a lithospheric boundary within the Superior province and terminating at the Superior-Yavapai Province boundary, reflects the upper mantle signature (i.e. ‘plume trail’) associated with Laurentia's rapid southwards motion over the Keweenaw mantle plume, as deduced from paleomagnetic studies. The high conductivity of only this segment of the plume trail is thought to result from metasomatism and/or partial melting of the sulfide-rich basal lithospheric mantle, melting of which is the source for magmatic sulfide deposits in this part of the Superior Province. In supporting the position of the previously interpreted plume center, our interpretation concurs with previous research where an early formed rift geometry controlled by pre-existing Archean structures explains the lack of a classical three arm, solely plume-related rift configuration.

However, the origin of the N-S trending conductive anomaly in the lithospheric mantle, and overlying scattered elliptical crustal anomalies, as well as the comparable isolated conductive anomalies in the central east parts of the study area is debatable based on the available data. Although they similarly may represent (at least in part) regional effects of the Keweenaw mantle plume localized by basement structures, hydrothermal fluid flow and magma emplacement into the crust in the Archean is proposed as a more likely alternative origin.

## METHODS

### The magnetotelluric method

The MT methodology uses measurements of time-varying and mutually orthogonal components of electromagnetic (EM) fields at the Earth's surface to image the subsurface resistivity structure [52,53]. Penetration depths of the EM fields increase with the increasing resistivity of the Earth and the period of EM field variations. The MT data used in this study were collected in the 1994–2000 Lithoprobe Western Superior Transect project in Canada [74] and the EarthScope USArray MT Program (2006–2018) in the United States [16]. As shown in Fig. 1, the EarthScope USArray MT data were acquired on a quasi-regular grid, while those of Lithoprobe were collected along several transects. Based on the spatial distribution and data quality, data from 169 long-period MT sites with periods of ∼10–20 000 s were selected, which is sufficient to probe the resistivity structure of the entire lithosphere [52,53,70].

### Inversion

3D MT inversions were carried out using ModEM [75,76] to fit the full impedance tensor (**Z**) and vertical magnetic field transfer functions (VTFs), also known as ‘tippers’. For the impedance, a full range of periods from 10–20 000 s were used, while for the VTFs, long periods of data (>7300 s) are excluded due to possible external source bias [77]. To reduce the calculation time and simultaneously ensure the model resolution, five periods per decade were used. To further reduce the calculation time and consider the possible influences from external structures (e.g. the oceans) that cannot be constrained by our data set, the nested model grid approach was used following previous studies [70]. Data of poor quality were excluded from the inversion.

The fine model embedded in the multi-gridded coarse model is of dimension 116 × 106 × 60 in x, y and z directions with a uniform grid-cell size of 8 km horizontally. The vertical grid-cell size is 50 m in the first layer and increases logarithmically by a factor of 1.15 to 1400 km, which is also the grid setting for the coarse model of the nested approach. The horizontal grid size in the coarse model in the multi-grid is 12 km, and it is padded logarithmically at the data boundary by a factor of 1.3, resulting in 1500 km between the model boundary and data boundary in each direction. For each model, 12 air layers were added to provide the upper boundary condition.

A series of inversions were conducted using different data sets, ‘error floors’, model smoothing factors, and starting and prior model resistivity, with the details shown in the supplementary data (Figs S5–S8). For the preferred model, the parameters are as follows. The resistivity of the starting and prior model is 100 Ω m; Atlantic Ocean and Hudson's Bay in the coarse model were set to be 0.3 Ω m. For the error floors, |Z_xy_ × Z_yx_|^1/2^ × 5% was set for all impedance components and a constant value of 0.05 was chosen for VTFs. The model smoothing was set to 0.2 with two passes in each direction. The impedance data were inverted first and a model was obtained with 218 iterations. Using this as the starting model, the inversion was restarted and the impedance and VTFs were jointly fitted. The preferred solution was obtained after an additional 146 iterations with a normalized root-mean-square (nRMS) misfit of 2.33. The good data fit of phase and apparent resistivity is shown in Figs S3 and S4, respectively.

## Supplementary Material

nwae239_Supplemental_File
