# Supplementary material for: Mantle plume trail beneath the ca. 1.1 Ga North American Midcontinent Rift revealed by magnetotelluric data
Source: Natl Sci Rev. 2024 Jul 13;11(8):nwae239. doi: 10.1093/nsr/nwae239 (PMC11334720; doi:10.1093/nsr/nwae239)
Supplement: nwae239_Supplemental_File [file nwae239_supplemental_file.pdf]

Supplementary data

Mantle plume trail beneath the ca. 1.1 Ga North American Midcontinent rift revealed by magnetotelluric data

Wule Lin[1,2,3], Adam Schultz[4], Bo Yang[5,6], Lyal B. Harris[7], and Xiangyun Hu[*] [1]

[1] Hubei Subsurface Multi-scale Imaging Key Laboratory, School of Geophysics and Geomatics, China University of Geosciences, Wuhan 430074, China

[2] Department of Ocean Science and Engineering, Southern University of Science and Technology, Shenzhen 518005, China

[3] Advanced Institute for Ocean Research, Southern University of Science and Technology, Shenzhen 518005, China

[4] College of Earth, Ocean, and Atmospheric Sciences, Oregon State University, Corvallis, OR 97331-5503, USA

[5] Key Laboratory of Ocean and Marginal Sea Geology, South China Sea Institute of Oceanology, Innovation Academy of South China Sea Ecology and Environmental Engineering, Chinese Academy of Sciences, Guangzhou 511458, China

[6] China-Pakistan Joint Research Center on Earth Sciences, CAS-HEC, Islamabad 45320, Pakistan

[7] INRS-ETE, 490 de la Couronne, Québec, QC, G1K 9A9, Canada June 25, 2024

This Supplementary data includes phase tensor analyses (Figure S1), 3-D model mesh (Figure S2), phase data fits (Figure S3), apparent resistivity fits (Figure S4), models obtained using various inversion parameters (Figures S5–S8), model sensitivity studies (Figures S9–S11), and details on the estimated fraction of graphite and sulfide minerals to produce anomalies (Figure S12).

1. Phase tensor analysis

The galvanic distortion of regional electrical fields caused by near-surface local heterogeneities must be accounted for in the application of the MT method [1] although it is arguable whether modern 3-D inverse modeling methods are as subject to its impacts as older, 1-D and 2-D methods. Nevertheless, we consider the impact of such distortions here, where distortion-free phase tensors were used in the data analysis [2]. As shown in Figure S1, a phase tensor can be depicted as an ellipse with Φ_{max} and Φ_{min} representing the major and minor axes respectively, with color representing phase $\sqrt{\Phi_{min}\Phi_{max}}$ that indicates the conductivity changes with depth.

The predominant high phase (> 60°) in Figure S1 indicates generally

* Corresponding author. E-mail address: xyhu@cug.edu.cn

increasing conductivity at great depth. Assuming the average resistivity is 100 Ω.m, periods of 33, 528, and 4673 s would represent the resistivity structure of the lower crust, lithospheric mantle, and asthenospheric mantle, respectively. Therefore, the high phase in northerly areas indicates a conductive upper mantle, which correlates well with our preferred model (Figure 3), especially when considering the resistivity values obtained. In contrast, the increment of conductivity in southern areas is generally slow, which may be caused by the slowly increasing temperature alone; at great depth (corresponding to 4673 s), the high phase indicates high conductivity, which is in good agreement with the preferred model as well.

2. Inversion

Figure S2 shows the 3-D model mesh used for the inversion. Figure S5 shows the model obtained using impedance data only, yet the main features of this model are similar to the preferred model in the main text and more continuous horizontally. Using different model smoothing parameters (Figures S5 and S6), the main features are similar, but the nRMS is small when the smoothing factor set to 0.2 with 2 passes. The influence on the model from the error floor of VTFs is not significant (Figure S7). However, with the error floor (i.e. 0.03) the nRMS of impedance data increased by 10% from the impedance-only inversion and the preferred solution. Using different staring models the main features are similar also (Figure S8). Therefore, we concluded that the main features discussed in the main text are well resolved by the MT data.

3. Sensitivity test

Figure S9 shows forward modeling tests on the depth extent of the model that were constrained by the MT data. We manually modified the resistivity of the preferred model below several selected depths, viz. 150, 200, 240, and 270 km, to the resistivity of the stating model 100 Ω.m (top panel), and calculated the corresponding forward response. The bottom panel shows site-by-site changes in nRMS from the preferred model for each modified model. Figures S10 and S11 shows resolution tests for the conductor DC1 and MC, respectively.

4. Analysis of conductive phases

Figure S12 shows details on the estimated fraction of graphite and sulfide minerals.

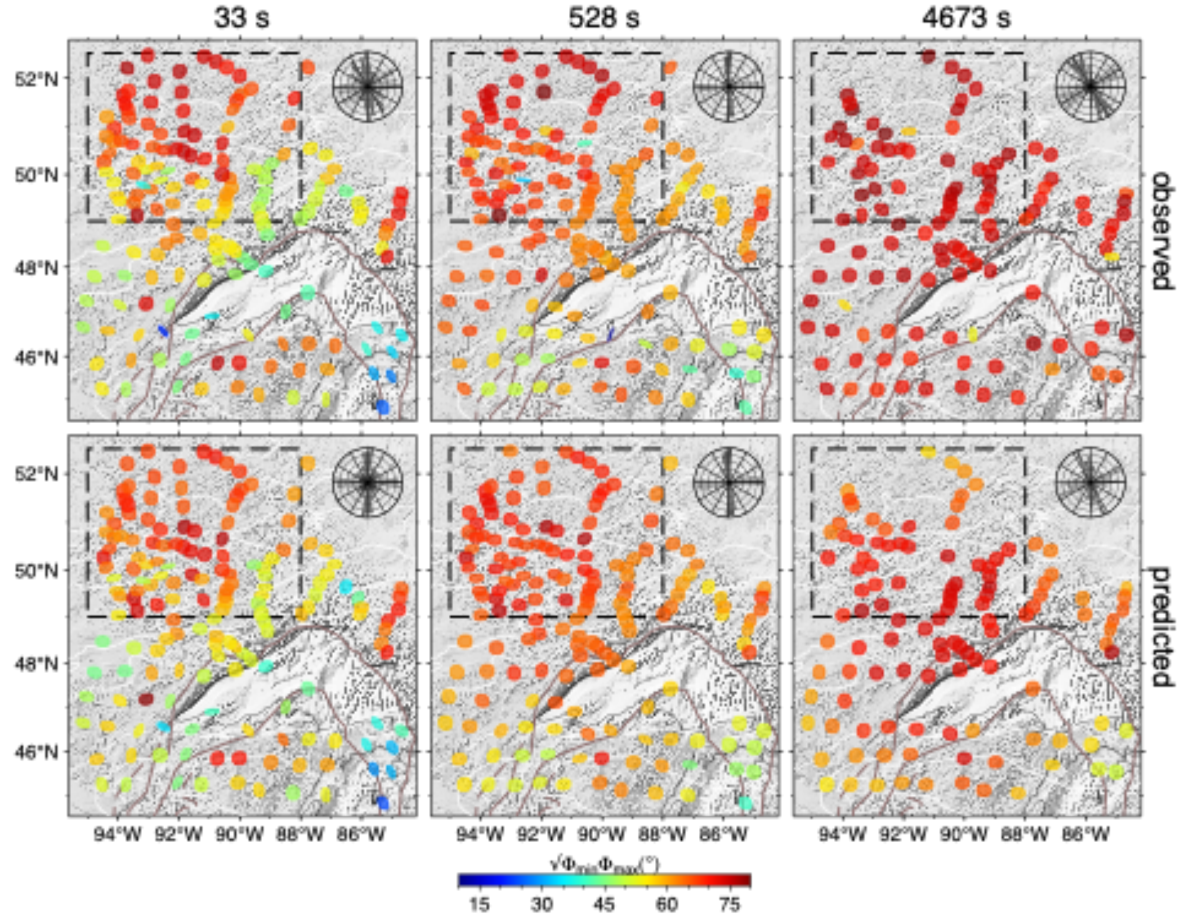

Figure S1: The observed (top) and predicted (bottom), i.e. calculated from the resistivity model inverted from the MT data, phase tensor ellipse of MT sites for periods of 33 s, 528 s and 4673 s with the base map showing tomography and terrane boundaries following Fig 1b. The ellipse sizes are normalized by the major axes Φ_{max} and the colors represent $\sqrt{\Phi_{min}\Phi_{max}}$. The rectangle outlined by dashed black lines shows the database that comprise the rose diagrams, which denotes the strike direction with 90° ambiguity. The good agreement of the observed and predicted phase tensor and strike direction indicates a good data fit, and thus the validity of the recovered model.

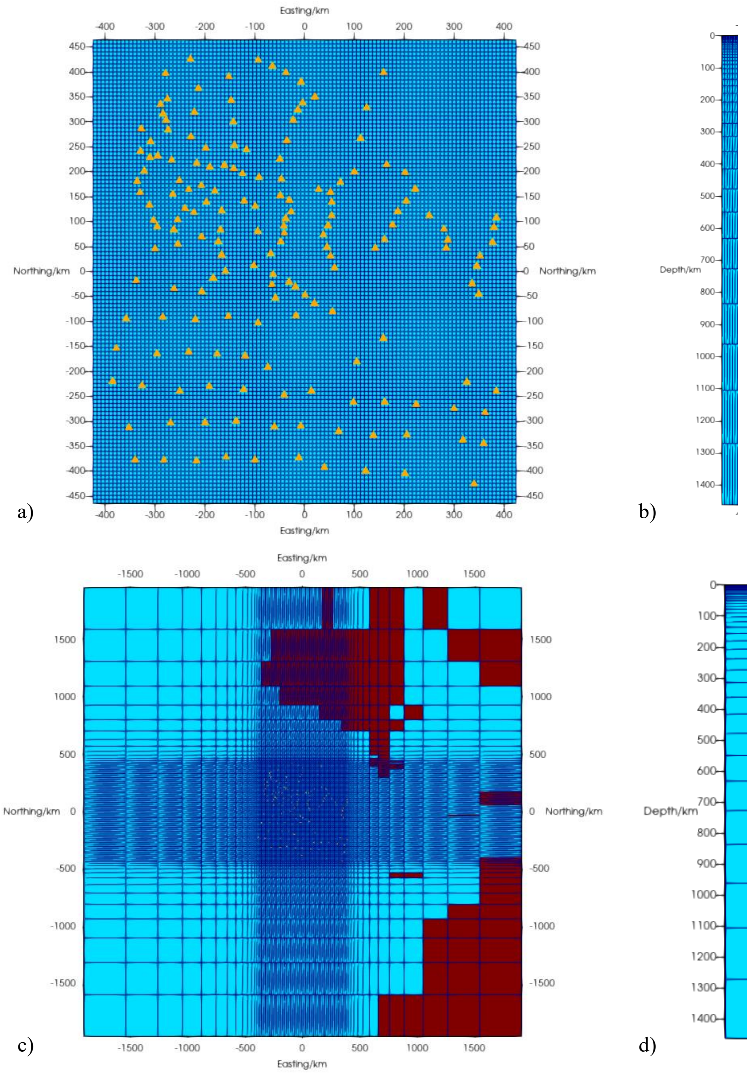

Figure S2 3-D model mesh used for the inversion. The fine mesh consisted of (a) 116 × 106 in N and E (respectively) with a regular cell size of 8 km. The horizontal grid size in the coarse model of (c) is 12 km and is padded logarithmically at the data boundary by a factor of 1.3, resulting in 1,500 km between the model boundary and data boundary in each direction. The vertical grid-cell size is 50 m in the first layer and increases logarithmically by a factor of 1.15 to 1,400 km for both (b) fine and (d) coarse mesh. The red area indicates the oceanic region with a resistivity of 0.3 Ω.m, which comes from ETOPO1 (https://www.ngdc.noaa.gov/mgg/global/).

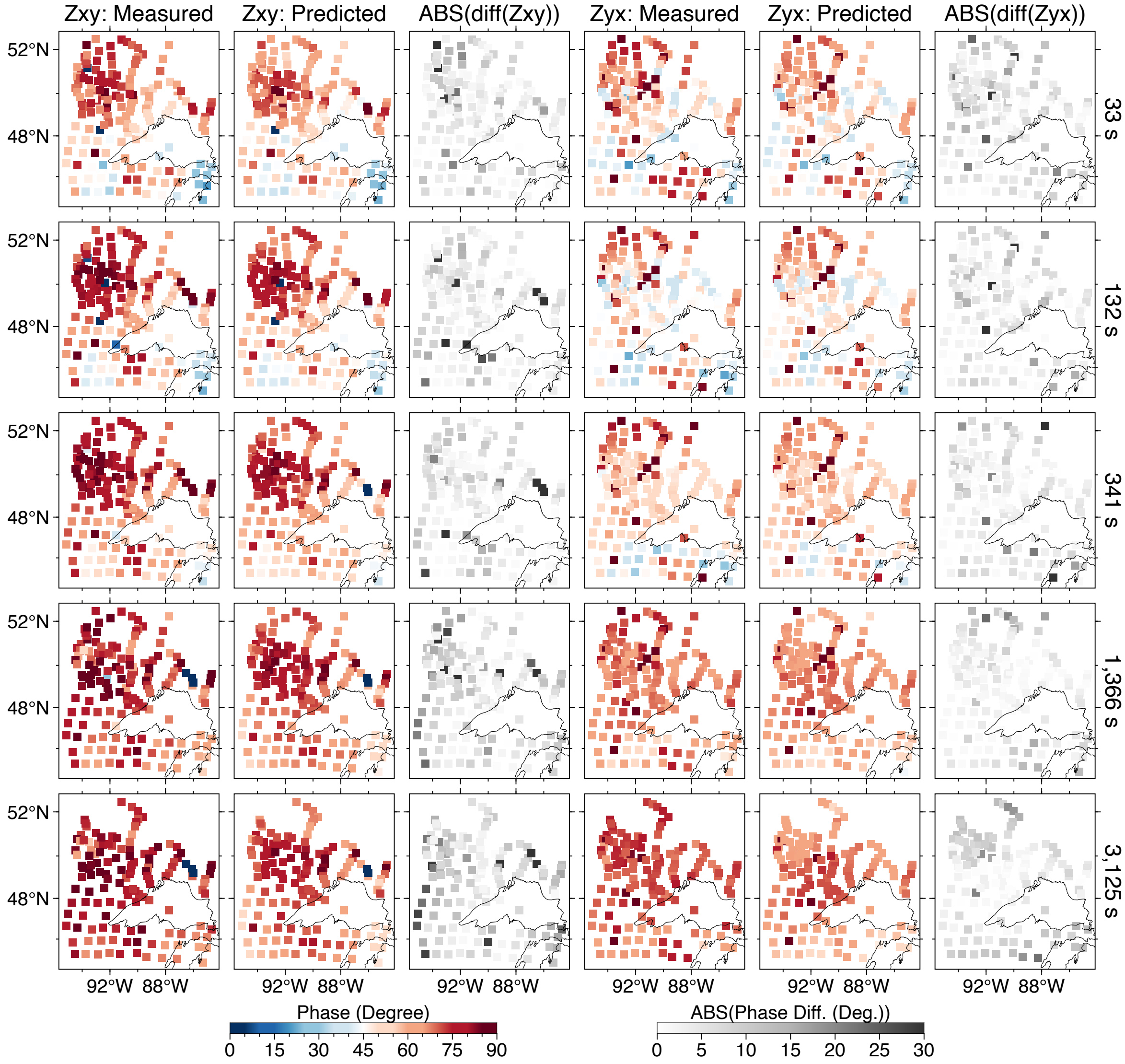

Figure S3: Measured and predicted phase angles along with their differences for each site at several periods for both TE and TM modes. These small differences indicate a good data fit.

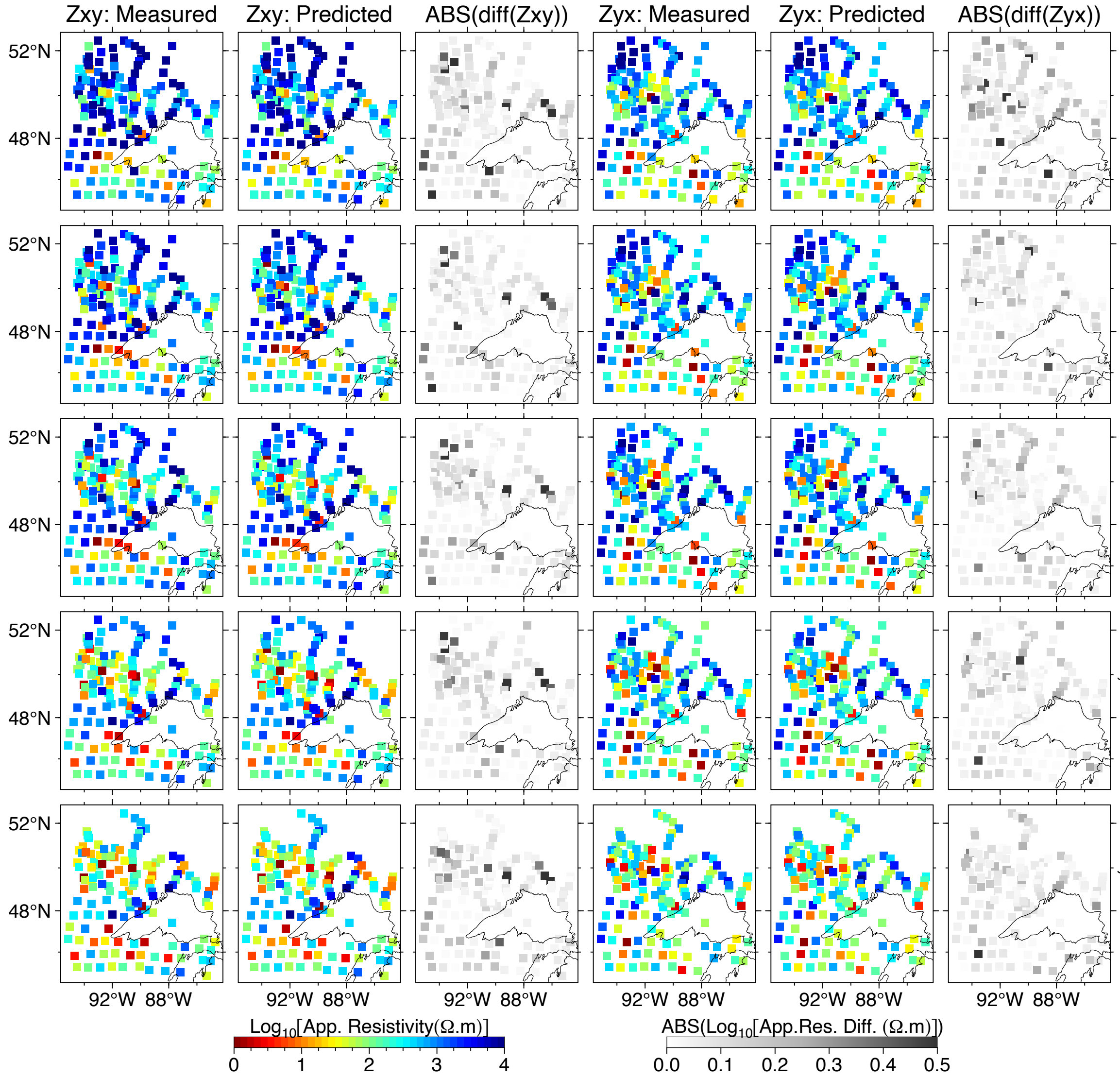

Figure S4: Same as Figure S3, but for apparent resistivity.

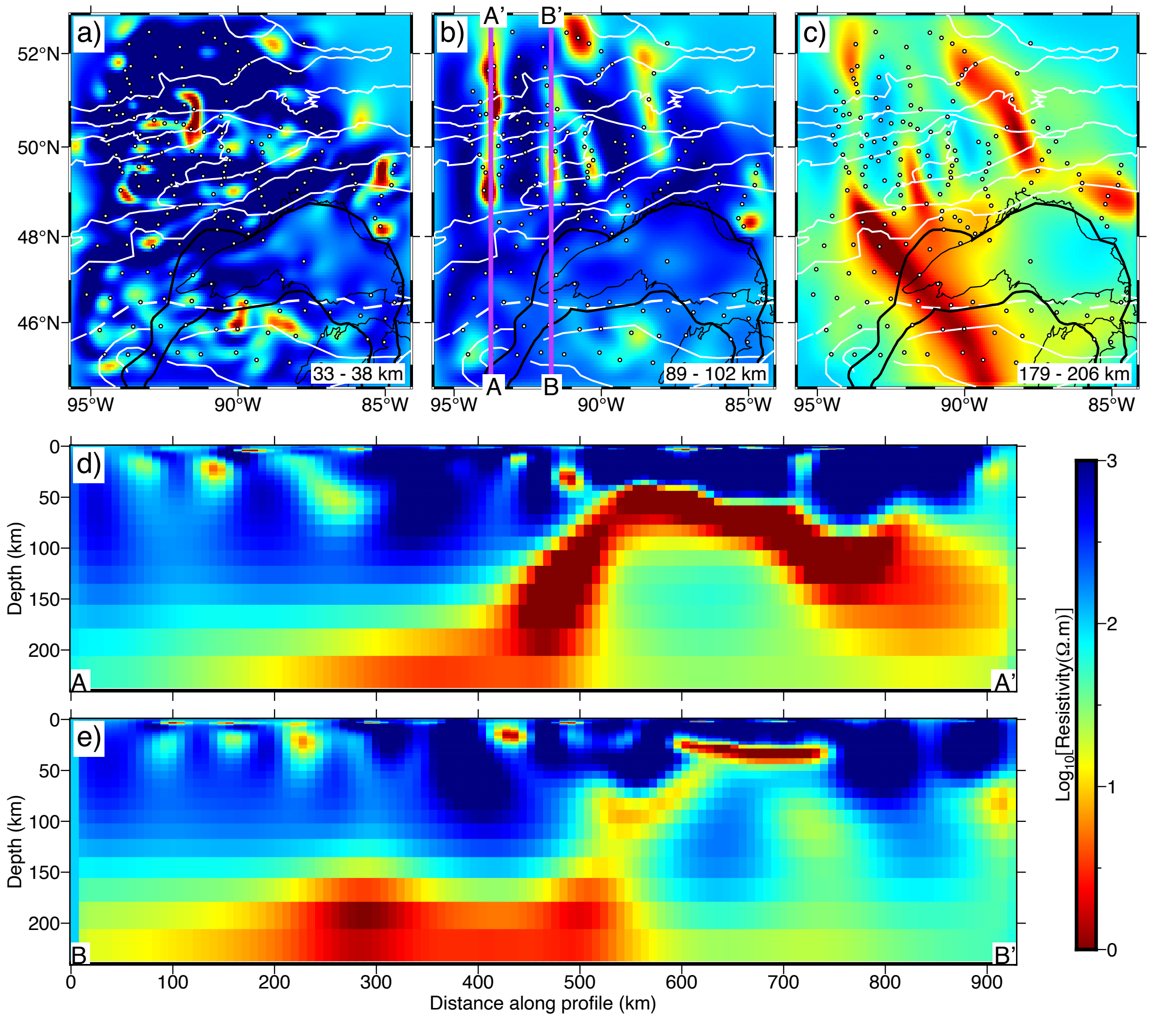

Figure S5: The resistivity model obtained using full impedance data alone. The other parameters were the same as for the preferred model.

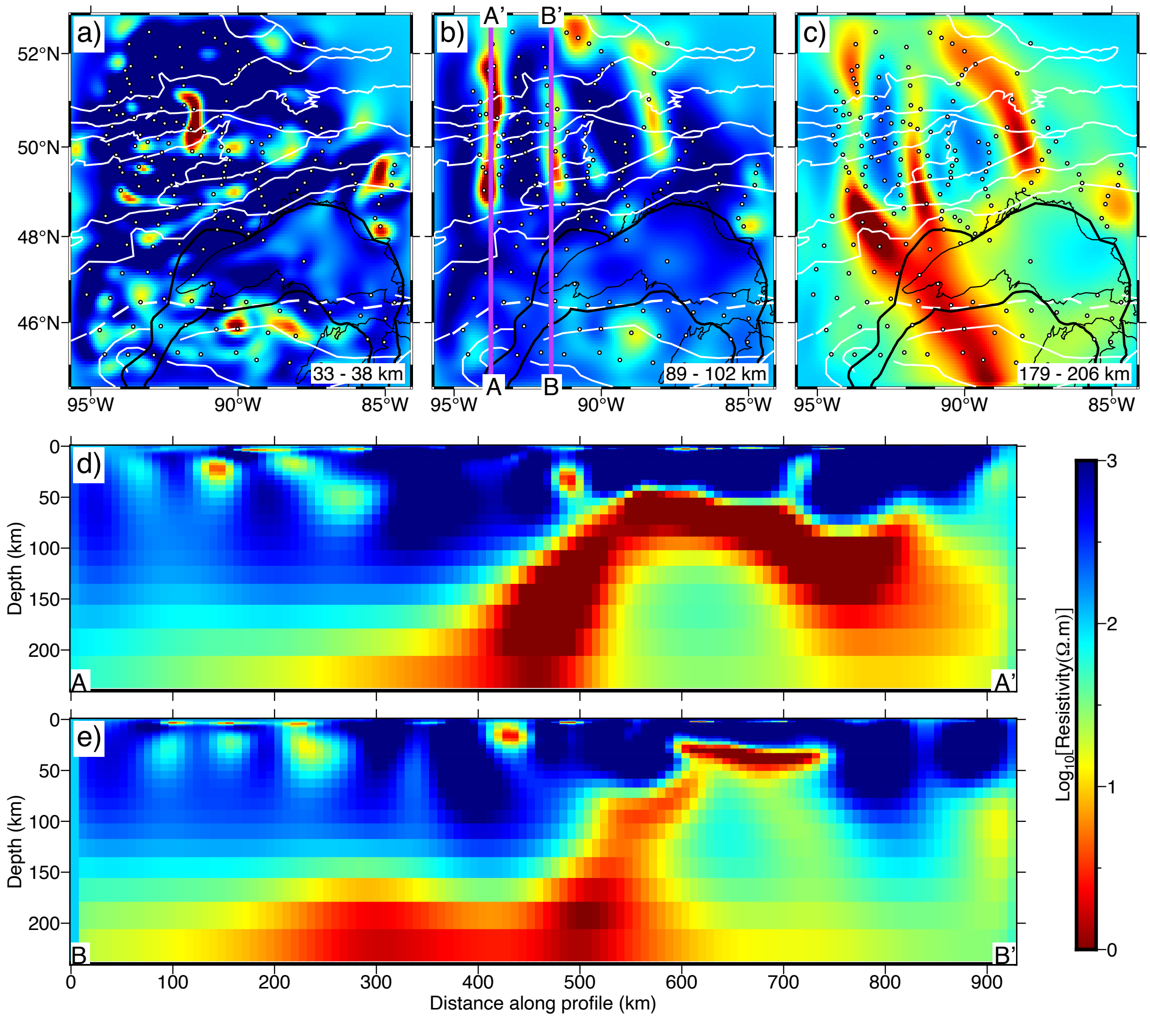

Figure S6: Same as Figure S5 except for the model smoothing parameter, which was set to 0.4, with 1 pass in each direction.

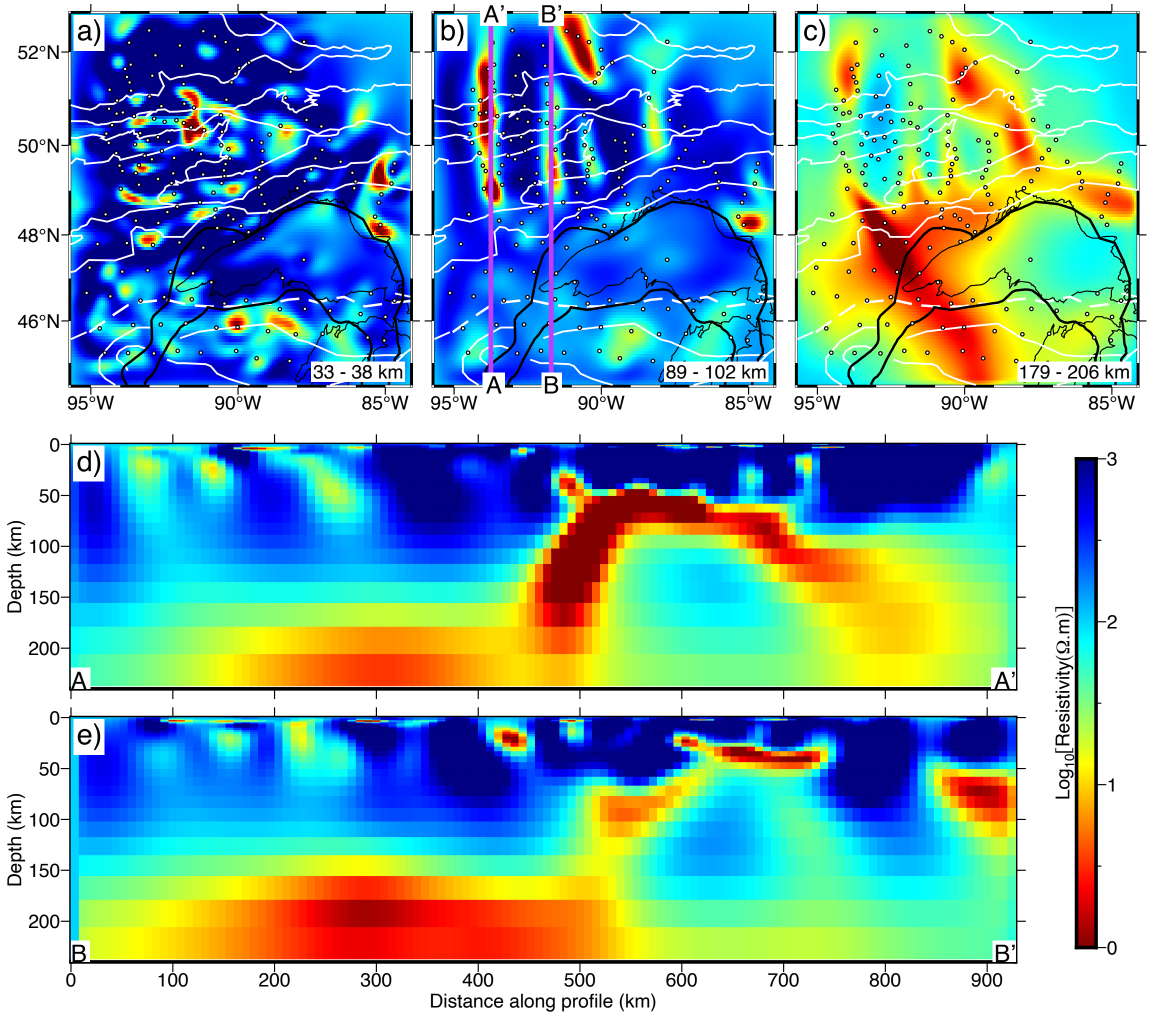

Figure S7: The resistivity model obtained using the same parameters as that for the preferred model in the main text apart from the data error for VTFs, which was a constant value of 0.03 (c.f 0.05).

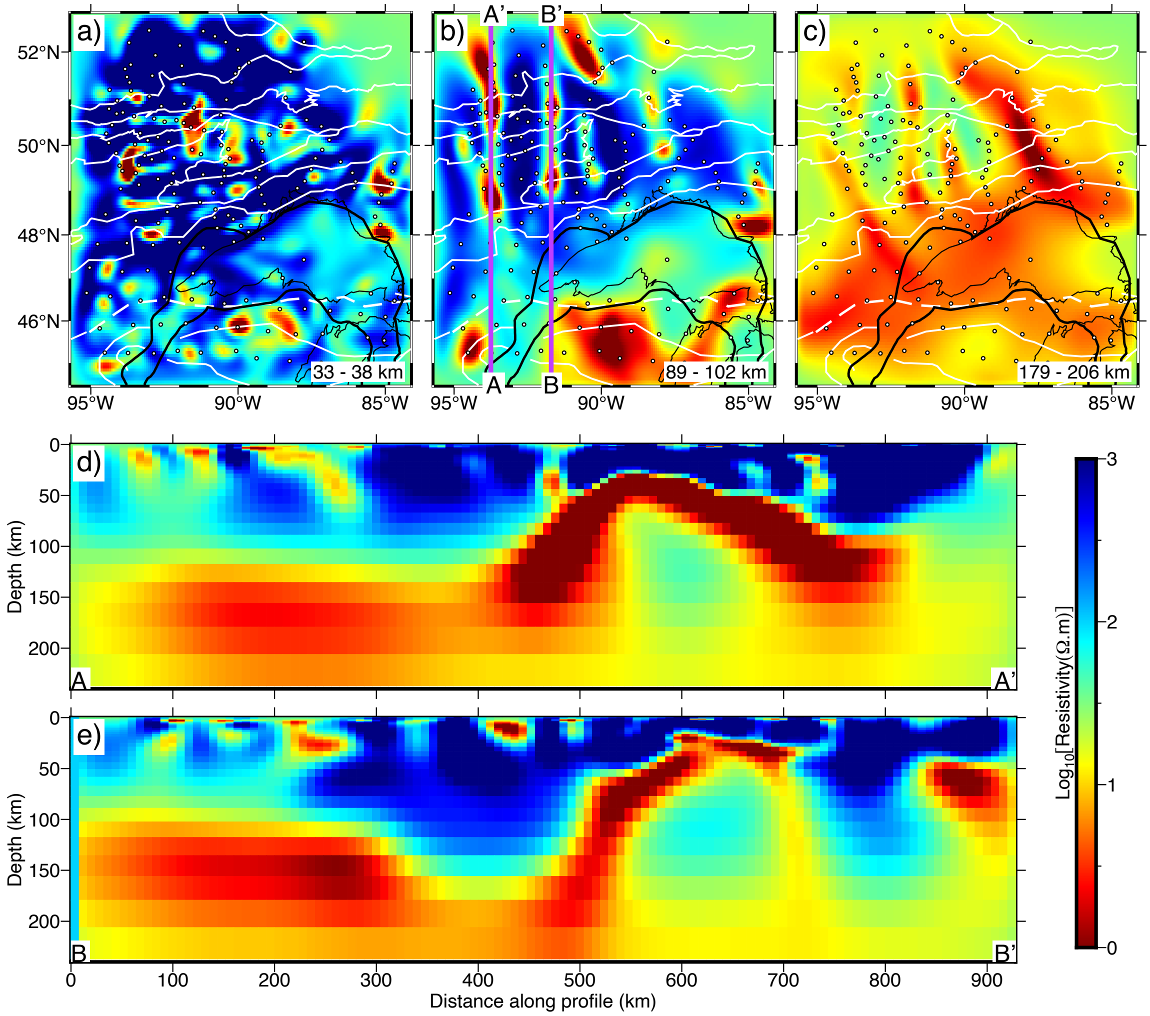

Figure S8: The resistivity model obtained using the same parameters as that for the preferred model in the main text apart from the staring resistivity, which was set to 30 Ω.m.

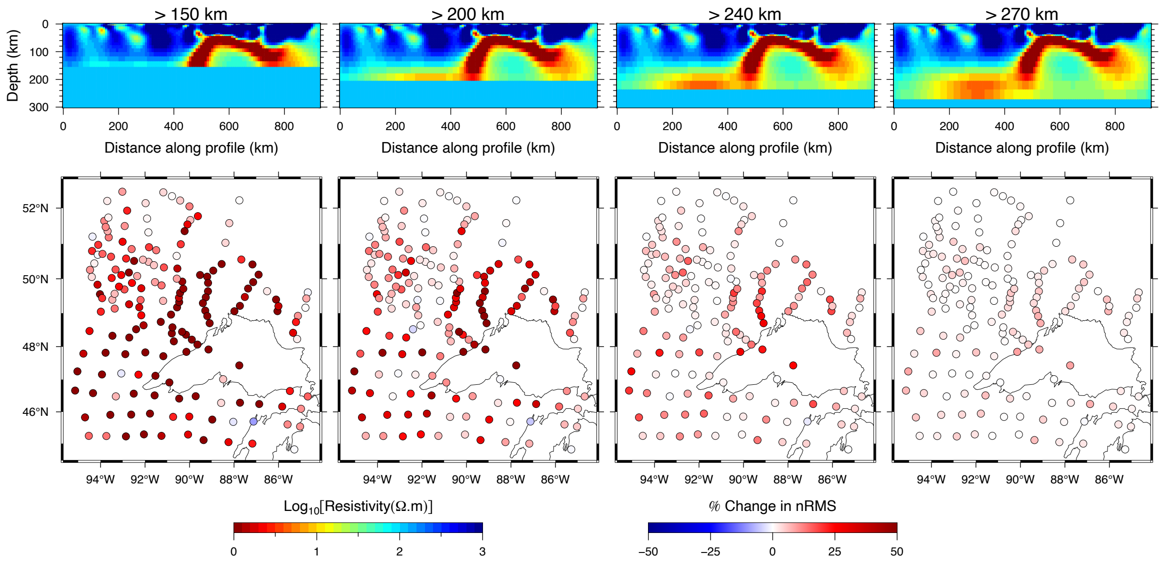

Figure S9: Forwarding modeling tests on the depth extent of the model that were constrained by the MT data. We manually modified the resistivity of the preferred model below several selected depths, viz. 150, 200, 240, and 270 km, to the resistivity of the stating model of 100 Ω.m (top panel), and calculated the corresponding forward response. The bottom panel shows site-by-site changes in nRMS from the preferred model for each modified model. Warm colors mean that the fit becomes worse while cold means better. The fit becomes obviously worse for the majority of sites for 150 and 200 km cases, indicating these structures, including the MC as shown in Figure 2c, are required by the dataset. The changes in nRMS become smaller for the deeper tests, especially for the regions with shallow conductive features due to the shallower penetration depth of electromagnetic waves. Note that all periods were included in the calculation; if the periods sensitive to deep resistivity changes only were to be calculated, the changes in nRMS would be larger. Therefore, we conclude that our dataset is sensitive to the resistivity structure at least to the depth of 240 km and even to 270 km, especially beneath resistive cratonic regions.

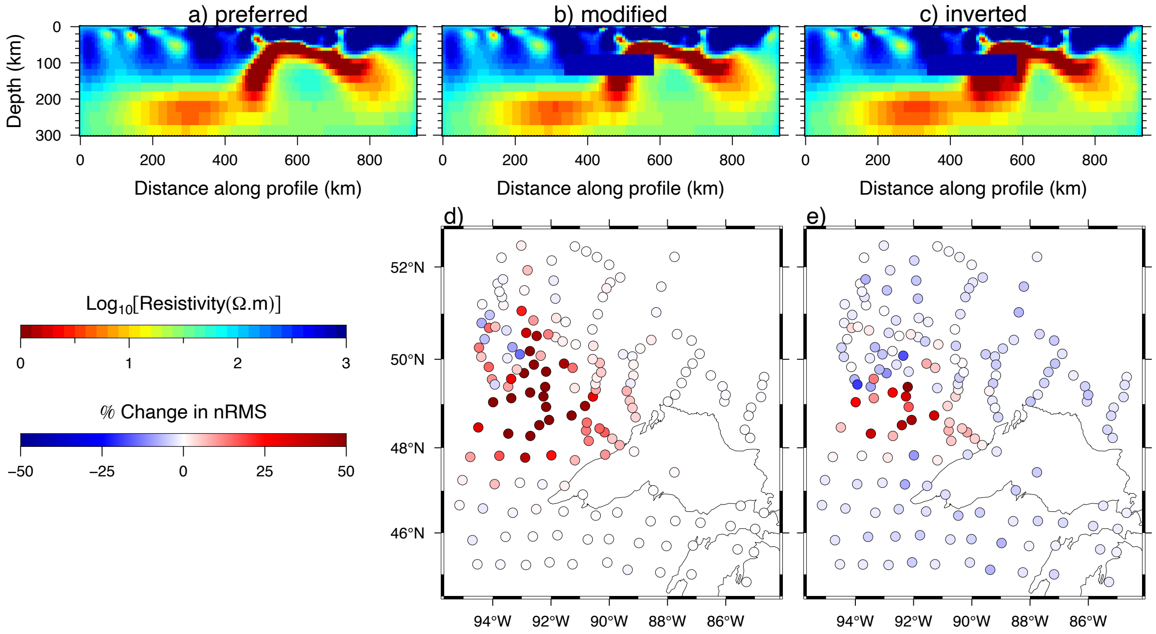

Figure S10: Resolution test for the conductor DC1. (b) shows the vertical location that was modified from the preferred model (a). The corresponding horizontal geometry that was modified is the narrow high conductivity DC1 as shown in Figure 2b. The replaced resistivity value was similar to that of surrounding area (700 Ω.m). As shown in (d), the fit of the data near the modified area becomes worse when the model was modified. We then restart the inversion keeping this modified resistivity fixed. As shown in (c), more conductive features around DC1 appear in order to compensate for the modified feature. Importantly, the nRMS around DC1 for the recovered model is still worse (e). Besides, modifying the extremely high conductivity alone (1 Ω.m) to other values (e.g. 3 or 10 Ω.m) also results in a worse data fit. We thus conclude that DC1 is required by the MT data.

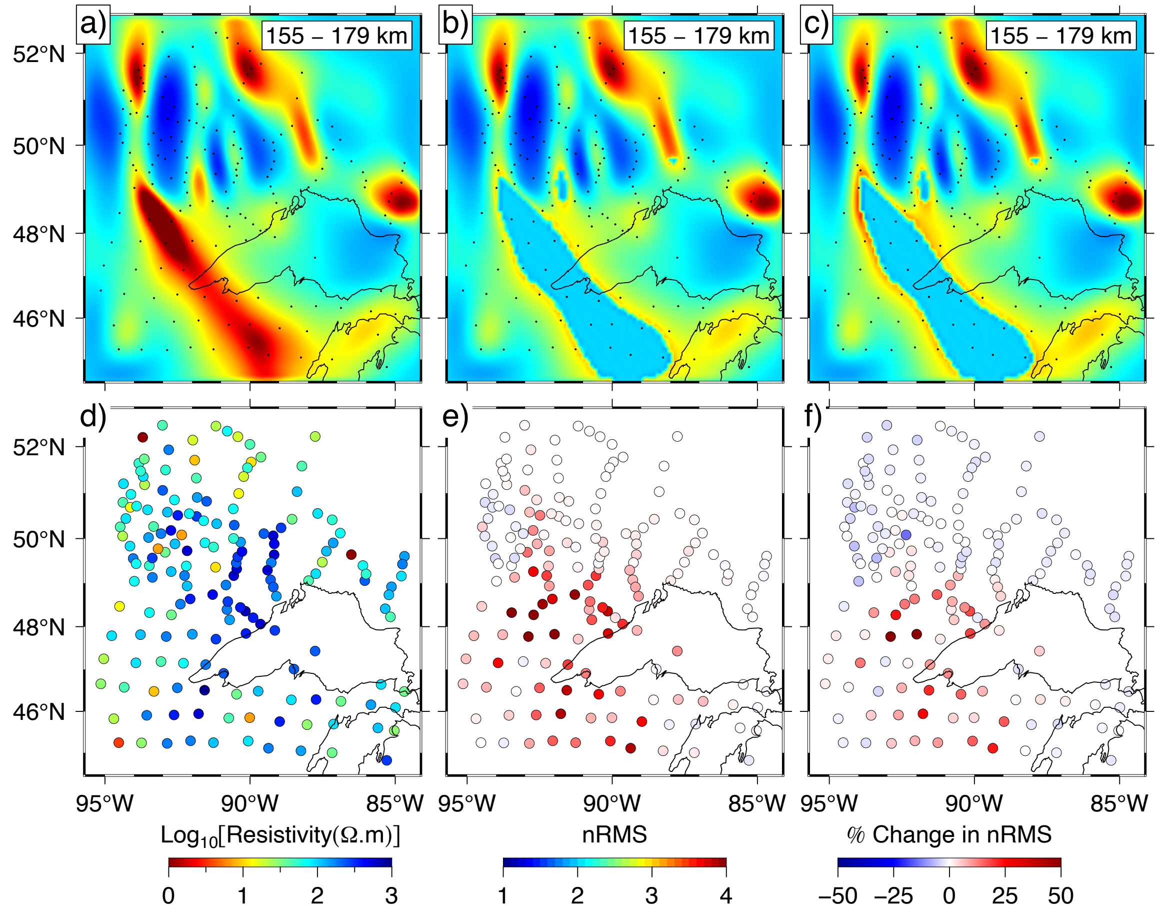

Figure S11: Resolution test for the conductor MC. (b) Horizontal geometry modified from the preferred model (a). The replaced resistivity value was similar to that of the starting model (100 Ω.m). (d) Site-by-site nRMS of the preferred model. (e) The fit of the data near the modified area becomes worse when the model was modified. The inversion was then restarted keeping this modified resistivity fixed. As shown in (c), more conductive features around MC appear in order to compensate for the modified feature. Importantly, the nRMS around MC for the recovered model is still worse. Besides, modifying the extremely high conductivity alone (1 Ω.m) to other values (e.g. 3 or 10 Ω.m) also results in worse data fit. We thus conclude that MC is required by the MT data.

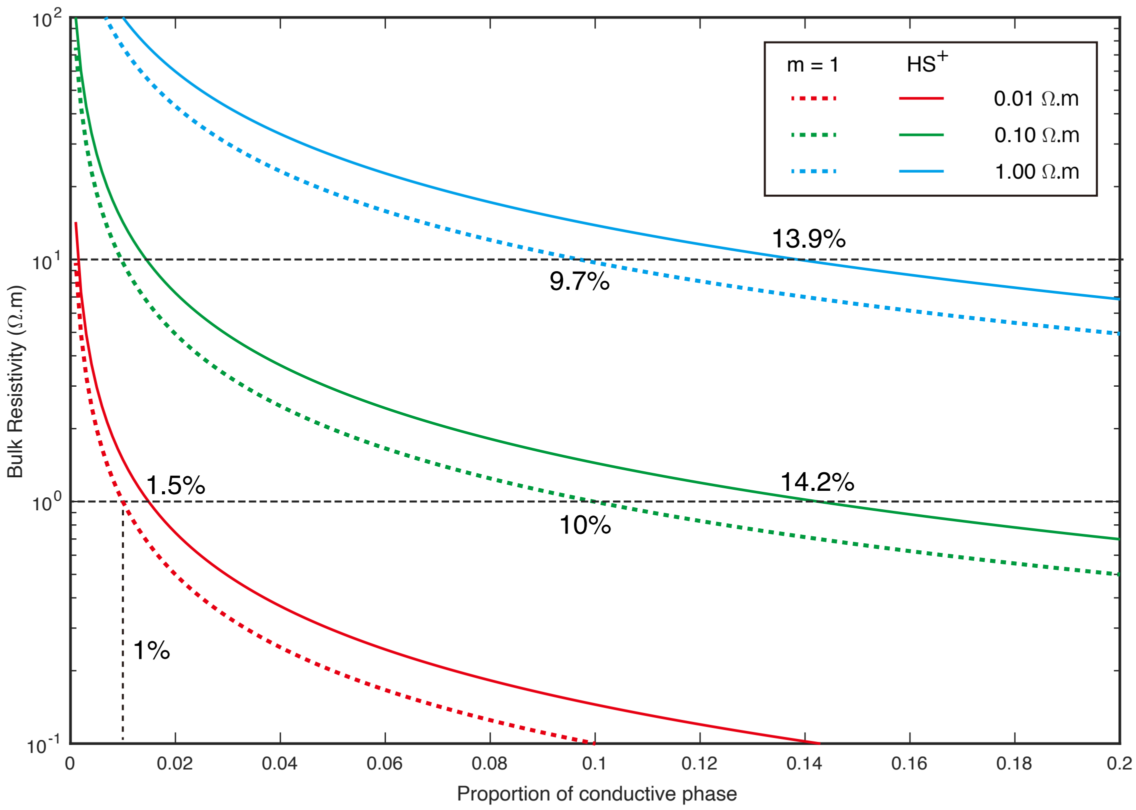

Figure S12: Bulk resistivity as a function of high conductive phase fraction, calculated using Hashin-Shtrikman upper bound (*HS*+) [3] and modified Archie's law [4] with a cementation factor $m = 1$, which denotes interconnected conductive phases. For the resistivity of the rock matrix, we used the highest temperature of lithosphere 1300°C and SEO3 law [5], resulting in resistivity of 300 Ω.m. With these choices, the least conductive fraction was obtained for the specific resistivity value of the conductor. The typical resistivity of graphite ranges between 0.1–10 Ω.m, while that of sulfides (Fe, Cu, Ni-rich) is generally less than 0.01 Ω.m even to ranges of 10^{-5}–10^{-3} Ω.m [6,7]. Considering the highest conductive of graphite, a reasonable fraction of ca. 1% would account for the conductor DC2, yet an unlikely amount is required to explain C1, DC1, and C2. However, a small amount of sulfide (ca. 1%) could account for C1 and DC1, and if 0.001 Ω.m is considered for the sulfide, this fraction would decrease to 0.1%. Besides, the resistivity of graphite could be much low also e.g. < 0.001 Ω.m [8] and thus be able to explain the extremely high conductivity anomaly.

References

1. Chave AD, Jones AG. *The magnetotelluric method: Theory and practice*. Cambridge: Cambridge University Press, 2012, 1–544.

2. Caldwell TG, Bibby HM, Brown C. The magnetotelluric phase tensor. *Geophys J Int* 2004, **158**: 457–69.

3. Hashin Z, Shtrikman S. A variational approach to the theory of the effective magnetic permeability of multiphase materials. *J Appl Phys* 1962, **33**: 3125–31.

4. Glover PWJ, Hole MJ, Pous J. A modified Archie's law for two conducting phases. *Earth Planet Sci Lett* 2000, **180**: 369–83.

5. Constable S. SEO3: A new model of olivine electrical conductivity. *Geophys J Int* 2006, **166**: 435–7.

6. Duba A, Heikamp S, Meurer W, Mover G, Will G. Evidence from borehole samples for the role of accessory minerals in lower-crustal conductivity. *Nature* 1994, 367: 59–61.

7. Ducea MN, Park SK. Enhanced mantle conductivity from sulfide minerals, southern Sierra Nevada, California. *Geophys Res Lett* 2000, **27**: 2405–8.

8. Simpson F, Bahr K. *Practical magnetotellurics*. Cambridge: Cambridge University Press, 2005, 1–192.
